# Protective effects of hyperbaric oxygen and iloprost on ischemia/reperfusion-induced lung injury in a rabbit model

**DOI:** 10.1186/2047-783X-17-14

**Published:** 2012-06-07

**Authors:** Ş Bozok, G Ilhan, Y Yilmaz, Z Dökümcü, L Tumkaya, H Karamustafa, S Ozan Karakisi, Ş Ergene, E Şener

**Affiliations:** 1 Department of Cardiovascular Surgery, Recep Tayyip Erdoğan University Faculty of Medicine, Training and Research Hospital, Rize, Turkey; 2 Department of General Surgery, Rize State Hospital, Rize, Turkey; 3 Department of Pediatric Surgery, Recep Tayyip Erdoğan University Faculty of Medicine, Training and Research Hospital, Rize, Turkey; 4 Department of Histology and Embryology, Recep Tayyip Erdoğan University Faculty of Medicine, Training and Research Hospital, Rize, Turkey

**Keywords:** Hyperbaric oxygen, Iloprost, Ischemia-reperfusion, Lung, Rabbit

## Abstract

**Background:**

The role of multiorgan damage in the mortality caused by ischemic limb injury is still not clarified. The objective of this study was to examine the potential protective effects of hyperbaric oxygen (HBO) and iloprost (IL) therapy on lung damage induced by limb ischemia/reperfusion injury in a rabbit model, using both biochemical and histopathological aspects.

**Methods:**

Forty New Zealand white rabbits were randomly allocated into one of five study groups: HBO group (single session of HBO treatment); IL group (25 ng/kg/min infusion of IL); HBO + IL group (both HBO and IL); Control group (0.9% saline only); and a sham group. Acute hind limb ischemia-reperfusion was established by clamping the abdominal aorta for 1 h. HBO treatment and IL infusion were administrated during 60 min of ischemia and 60 min of reperfusion period. Blood pH, partial pressure of oxygen, partial pressure of carbon dioxide and levels of bicarbonate, sodium, potassium, creatine kinase, lactate dehydrogenase, and tumor necrosis factor alpha were determined at the end of the reperfusion period. Malondialdehyde was measured in the plasma and lung as an indicator of free radicals. After sacrifice, left lungs were removed and histopathological examination determined the degree of lung injury.

**Results:**

In the control group, blood partial pressure of oxygen and bicarbonate levels were significantly lower and creatine kinase, lactate dehydrogenase, malondialdehyde and tumor necrosis factor-α levels were significantly higher than those of the HBO group, IL group, HBO + IL group and sham group. Similarly, the malondialdehyde levels in the lung tissue and plasma levels were significantly lower in the treatment groups compared with the control group. The extent of lung injury according to the histological findings was significantly higher in the control group.

**Conclusions:**

These results suggest that both HBO and IL therapies and their combination might be effectively used in the prevention of lung injury after ischemia/reperfusion injury of the lower extremities.

## Background

Re-establishing perfusion after an ischemic period in a tissue worsens the initial ischemic injury. This process is known as ischemia/reperfusion injury (IRI) [1]. Surgical interventions that need temporary aortic cross clamping are followed by ischemia and reperfusion (I/R) of distal extremities. IRI may occur in an ischemic extremity or organ (local injury) or in distal parts far from ischemic areas. Intestinal I/R may be the source of proinflammatory mediators with the release of cytokines such as TNF-α, interleukin-1β and activation of polimorphonuclear cells, which play a major role in this injury [2]. In fact, TNF-α was reported as an essential cytokine in the inflammatory response after I/R, and as a pleiotropic cytokine that might trigger either cell death or proliferation. Free oxygen radicals at the very beginning of reperfusion are known to increase the harmful effect of injury [3]. Free radical-induced peroxidation of cell membrane macromolecules is an important element of IRI. Lipid peroxidation occurring during I/R is a chain reaction leading to the oxidation of polyunsaturated fatty acids that, in turn, disrupts the structure of biological membranes and produces toxic metabolites, such as malondialdehyde (MDA) [4].

Remote organ damage caused by I/R of the lower limb targets the lungs, a fact which is of major importance. I/R of the lower limb leads to pulmonary edema by means of pulmonary vasoconstriction, pulmonary hypertension and a rise in alveolar membrane permeability [2]. Various drugs have been reported to reduce IRI during aortic cross clamping [5,6]. Recently, studies have demonstrated that hyperbaric oxygenation (HBO) limits the consequences of IRI in various tissues [7,8]. It has been proposed that HBO affects factors contributing to the pathogenesis of IRI, including neutrophils, the endothelium, inflammatory mediators, lipid peroxidation and microvascular blood flow [8]. HBO treatment involves the inspiration of 100% oxygen intermittently for 60 to 90 min at a pressure higher than normal atmospheric pressure. Exposure to HBO increases the dissolved oxygen concentration in the arterial blood (pO_2_) and enhances the rate of diffusion of oxygen into poorly perfused tissues [9]. The neuroprotective effect of iloprost (IL), a stable prostacyclin analog, has received attention and has been used in some experimental studies. IL mimics the pharmacodynamic properties of prostacyclin, such as the inhibition of platelet aggregation, vasodilatation and cytoprotection [10]. Pretreatment with IL decreases pulmonary injury after ischemia [11].

The aim of the present study was to examine the potential protective effects of HBO and IL on lung damage induced by limb I/R in a rabbit model, using both biochemical and histopathological aspects.

## Methods

The experimental procedure was in accordance with the *Position of the American Heart Association on Research Animal Use*[12]. Animal care complied with the *Principles of Laboratory Animal Care* as formulated by the National Society for Medical Research and the *Guide for the Care and Use of Laboratory Animals* (National Institutes for Health publication No. 5377–3, 1996). The experimental study was approved by the Animal Research Ethics Committee of Rize University Medical Faculty. All animals were given 5 days of adaptation to their environment prior to experiments. The room temperature was kept between 28 °C to 30 °C.

### Experimental protocol

Forty New Zealand white male rabbits, weighing 2.5 to 3 kg, were randomly allocated into one of five study groups. The experiment was continued until eight animals in each group survived during the entire procedure and during the postoperative 4 h. The HBO group received a single session of HBO treatment the IL group received 25 ng/kg/min infusion of IL; the HBO + IL group received both HBO and IL; the control group received only 0.9% saline; and the fifth group was the sham group. HBO treatment and IL infusion were administrated during 60 min of ischemia and 60 min of reperfusion in the treatment groups.

Initial anesthesia was achieved with intramuscular ketamine (50 mg/kg) and xylazine (5 mg/kg) without endotracheal intubation; then followed by 25 mg/kg fractionally in order to allow the animals to have spontaneous respiration. All animals received a similar volume of maintenance fluids (0.9% sodium chloride, 20 ml/h) for the whole procedure. An arterial catheter (20-gauge) was placed in an ear artery to monitor blood pressure (Petas KMA 800, Ankara, Turkey). Each animal received 150 U/kg heparin intravenously for anticoagulation. Animals were placed in the supine position. The infrarenal abdominal aorta was exposed through a midline laparotomy incision and a transperitoneal approach, with the abdominal contents reflected to the right. The aorta was exposed from the left renal artery down to the aortic bifurcation. In all animals, except the sham controls (n = 8), the aorta was cross-clamped just proximal to the aortic bifurcation using atraumatic arterial bulldog clamps (Vascu-statt, Scanlan International, St. Paul, MN, USA). The clamp was removed after 60 min and restoration of blood flow was verified visually. Four hours after the intervention, all animals were humanely killed by a lethal cardiac injection of pentobarbital (100 mg/kg), immediately after which a median sternotomy was used to extract lung tissues for histopathological examination by light microscopy and biochemical investigations by MDA assay. The left lungs were removed, fixed with 10% tamponated formalin and stored for 24 hours. A sagittal section was obtained at the level of the hilum. These tissues were fixed again with 10% formalin for 2 days. Three animals died during the procedure, one in the HBO group during anesthetic induction, one in the IL group due to surgical intervention, and one in the sham group due to hemorrhage. They were excluded from the study.

### Hyperbaric oxygen treatment procedure

Animals received HBO therapy in an animal monoplace chamber during 60 min of ischemia and 60 min of reperfusion period at 2.5 atmospheric pressure. Before pressurization, 100% medical oxygen was flushed through the chamber for 10 min to displace ambient air. The oxygen pressure was then increased slowly and reached 2.5 ATA in 5 min. The chamber was ventilated during HBO therapy to avoid carbon dioxide (CO_2_) accumulation. Oxygen content was monitored continuously and maintained ≥98.5% (Nellcor:Draeger, Hemel Hempstead, Hertfordshire, UK). The concentration of CO_2_ was not allowed to rise above 0.1%. An environmental control system maintained the inner temperature and relative humidity at 25 ± 1 °C and 50 ± 20%, respectively. After 120 min at 2.5 ATA, the chamber was decompressed to normal atmospheric pressure in 5 min.

### Biochemical analysis

Blood pH, pO_2_ (mmHg), pCO_2_ (mmHg), bicarbonate (HCO_3_; mmol/L), sodium (Na^+^), potassium (K^+^), creatine kinase (CPK; IU/L), lactate dehydrogenase (LDH; IU/L) and TNF-α (pg/mL) values were determined 1 h after the onset of ischemia and 1 h after the onset of reperfusion. Blood pH, pO_2_, pCO_2_, HCO_3_, Na^+^ and K^+^ values were determined using a blood gas analyzer (Ciba Corning Blood Gas Analyzer Model 860, Ciba Corning Diagnostics Corp., Irvine, CA, USA). Plasma CPK and LDH levels were measured in lithium heparinized plasma using automated enzyme reactions (automated analysis for Hitachi System 717, Boehringer Mannheim Diagnostica, Mannheim, Germany).

### TNF-α assay and malondialdehyde assay

TNF-α assays were performed according to the method described by Pizarro *et al.* using the quantitative sandwich enzyme immunoassay technique (Quantikine M, R&D Systems, Minneapolis, MN, USA) [13]. Lipid peroxidation in the lung tissue was evaluated by measuring the level of MDA, which is the end product of lipid peroxidation. MDA was measured in plasma and the lung as an indicator of free radicals. Plasma and tissue (lung) MDA levels were determined with spectrophotometry using thiobarbituric acid-reactive substances [14].

### Histopathological assay

After standard tissue preparation, 5 μm tissue sections were obtained. The specimens were stained with hematoxylin-eosin and examined with light microscopy (Eclipse E200; Nikon, Tokyo, Japan). All histopathological changes were detailed in each lung tissue, including inflammatory cell infiltration, alveolar edema, congestion and preservation of the alveolar septum. At least two different sections were explored in each specimen. The same pathologist, who was blinded to the study, assigned a score of 0 to 4 on the basis of congestion, interstitial edema, polymorphonuclear leukocyte infiltration and alveolar hemorrhage as follows: 0, normal histological appearance; 1, mild and focal changes; 2, moderate and multifocal changes; 3, marked and multifocal changes; 4, marked and diffuse changes.

### Statistical analyses

Data were analyzed using the Statistical Package for Social Sciences (SPSS) software (version 10.0 for Windows). All differences associated with a chance probability of 0.05 or less were considered statistically significant. All data are presented as mean ± SD. Statistical analysis was performed using analysis of variance (ANOVA) test. One-way ANOVA test was followed by post-hoc Dunnett's test.

## Results

There was no statistically significant difference between the groups according to the preoperative, ischemia and reperfusion periods. Hemodynamic variables of all groups are shown in Table 1. Table 2 shows mean values and SDs of pH, pO_2_, pCO_2_, HCO_3_. Table 3 shows Na^+^, K^+^, CPK, LDH, plasma MDA and lung MDA levels. Pre- and postischemic mean arterial pressure was minimally lower in HBO and IL groups when compared with controls. Blood pH, PO_2_, and HCO_3_ levels of the medicated groups were significantly higher than the levels of the control group. Plasma CPK, plasma MDA and lung MDA levels were significantly lower in the HBO group, IL group, HBO + IL group and sham group than those of the control group. Administration of HBO and/or IL suppressed MDA increase in plasma (*P* <0.01) and in the lung tissue (*P* <0.01). Compared with the control group, serum TNF-α levels in the treatment groups and the sham group were found to be significantly lower in the reperfusion period. Furthermore, combined therapy of HBO and IL was shown to be more effective than either treatment given singly (Table 4).

**Table 1:** Hemodynamic parameters determined at the end of the reperfusion period

**Groups**	**Mean arterial pressure (mmHg)**	**Heart rate (bpm)**	**Central venous pressure (mmHg)**	**Cardiac rhythm**
**HBO group**	64 ± 10	132 ± 25	6 ± 2	Sinus rhythm
**IL group**	68 ± 12	138 ± 20	8 ± 3	Sinus rhythm
**HBO + IL group**	66 ± 8	136 ± 18	7 ± 3	Sinus rhythm
**Control group**	74 ± 10*	154 ± 28	6 ± 2	Sinus rhythm
**Sham group**	65 ± 6	134 ± 15	7 ± 2	Sinus rhythm

*Significantly higher than the treatment group by analysis of variance (*P* <0.05). Bpm: beats per minute; HBO: hyperbaric oxygenation; IL: iloprost.

**Table 2:** Blood pH, partial pressure of oxygen, partial pressure of carbon dioxide and bicarbonate levels determined at the end of the reperfusion period

**Groups**	**pH**	**pO**_**2**_**(mmHg)**	**pCO**_**2**_**(mmHg)**	**HCO**_**3**_**(mmol/L)**
**HBO group**	7.23 ± 0.32	96.80 ± 7.02	42.60 ± 4.50	16.42 ± 5.3
**IL group**	7.24 ± 0.40	95.78 ± 6.35	43.75 ± 5.30	15.44 ± 7.2
**HBO + IL group**	7.22 ± 0.38	97.09 ± 7.06	42.09 ± 4.08	16.75 ± 5.5
**Control group**	7.14 ± 0.10*	76.10 ± 6.90*	49.84 ± 6.3**	9.12 ± 6.8*
**Sham group**	7.23 ± 0.22	94.05 ± 4.05	44.02 ± 4.20	17.56 ± 6.2

*Significantly lower than the treatment groups by analysis of variance (*P* <0.05); **significantly higher than the treatment groups by analysis of variance (*P* <0.05). HBO: hyperbaric oxygenation; HCO_3_: bicarbonate; IL: iloprost: pCO_2_: partial pressure of carbon dioxide; pO_2_: partial pressure of oxygen.

**Table 3:** Blood sodium and potassium levels, plasma lactate dehydrogenase and malondialdehyde levels and lung tissue malondialdehyde levels determined at the end of the reperfusion period

**Groups**	**Sodium**	**Potassium**	**Lactate dehydrogenase (IU/L)**	**Plasma malondialdehyde (μmol/L)**	**Lung malondialdehyde (μmol/L)**
**HBO group**	141.36 ± 4.3	4.3 ± 1.2	622.22 ± 146.5	21.60 ± 2.3	238.04 ± 36.11
**IL group**	140.52 ± 3.8	4.6 ± 1.1	627.44 ± 134.2	24.19 ± 2.8	240.06 ± 32.03
**HBO + IL group**	142.40 ± 4.1	4.3 ± 1.0	626.50 ± 138.3	22.30 ± 2.2	236.90 ± 25.02
**Control group**	146.32 ± 4.4	5.2 ± 0.9	646.83 ± 167.2*	30.12 ± 1.4*	252.07 ± 46.21*
**Sham group**	143.60 ± 7.2	4.4 ± 1.2	625.10 ± 144.3	23.63 ± 0.13	238.08 ± 35.39

*Significantly higher than the treatment groups by analysis of variance (*P* <0.05). HBO: hyperbaric oxygenation; IL: iloprost.

**Table 4:** Serum tumor necrosis factor alpha values determined preoperatively, on the first hour of ischemia and on the first hour of the reperfusion period

**Groups**	**Preoperative (pg/mL)**	**First hour of ischemia (pg/mL)**	**First hour of reperfusion (pg/mL)**
**HBO group**	12 ± 2.2	362 ± 41.9	152 ± 23.48
**IL group**	9 ± 2.4	346 ± 47.6	147.25 ± 22.78
**HBO + IL group**	14 ± 2.1	314 ± 53.1	144.88 ± 32
**Control group**	10 ± 2.1	654 ± 40.2*	484.38 ± 40.45
**Sham group**	8 ± 2.03	168 ± 11.9	46.25 ± 8.35
***P***	0.884	0.001	0.001

*Significantly higher than the treatment groups by analysis of variance (*P* <0.05). HBO: hyperbaric oxygenation; IL: iloprost.

**Figure 1:**
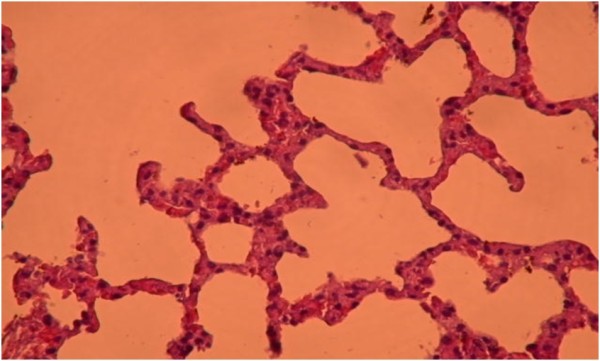
**Healthy lung tissue without ischemic injury (hematoxylin and eosin × 200).** Hyperbaric oxygenation **+** iloprost group.

**Figure 2:**
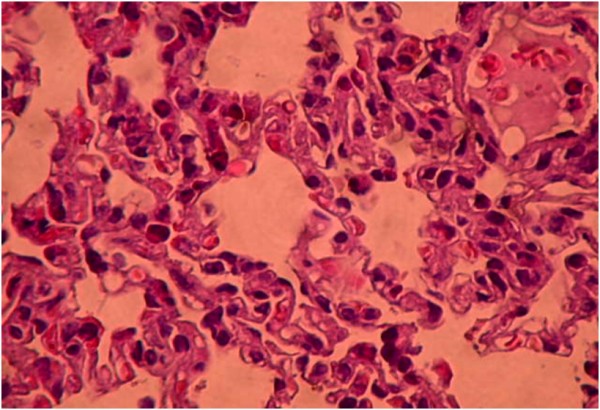
**Moderate degree of ischemic lung injury characterized by interstitial septal thickening and bleeding areas (+) (hematoxylin and eosin × 200).** Hyperbaric oxygenation group.

**Figure 3:**
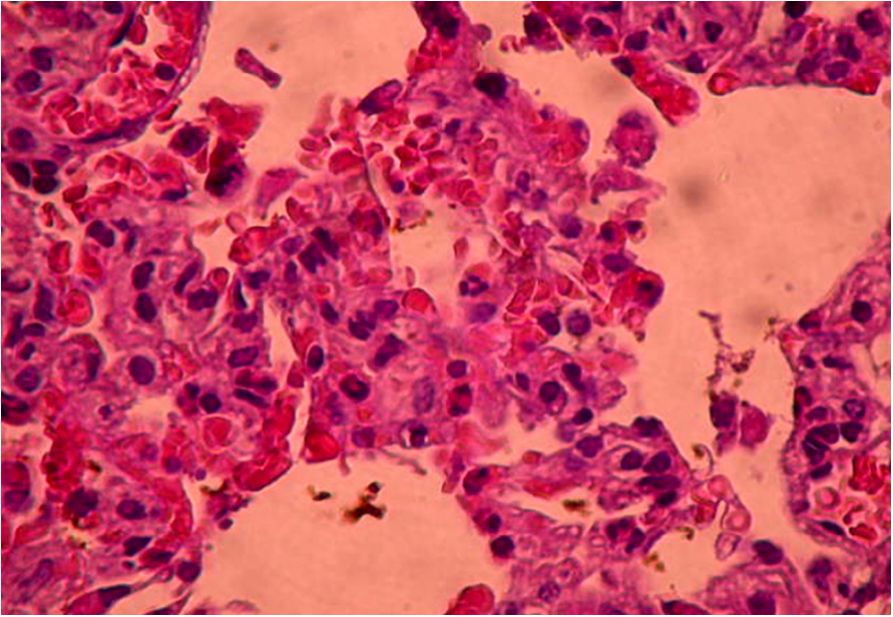
**Moderate degree of ischemic lung injury characterized by interstitial septal thickening and bleeding areas (+++) (hematoxylin and eosin × 200).** Iloprost group.

**Figure 4:**
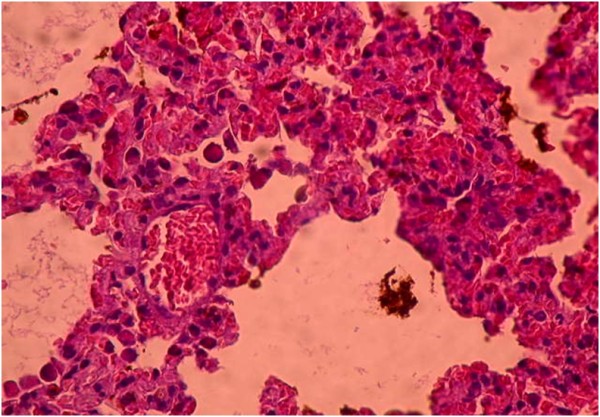
**Diffuse, severe ischemic lung injury characterized by bleeding, edema and interstitial congestion (hematoxylin and eosin × 200).** Control group.

Histopathological findings are summarized in Table 5. Diffuse severe ischemic lung injury, characterized by bleeding, edema and interstitial congestion, was significantly lower in the HBO group, IL group, HBO + IL group and sham group (*P* <0.01). In addition, the histopathological score was significantly lower (*P* <0.01) in the HBO group, IL group, HBO + IL group and sham group when compared with the control group (Figures 1,2,3 and 4). There was no significant difference between groups according to the other parameters.

**Table 5:** Histopathological results determined at the end of the reperfusion period

**Groups**	**Grade 0**	**Grade 1**	**Grade 2**	**Grade 3**	**Grade 4**
**HBO group (n = 8)**	1	3	3	1	-
**IL group (n = 8)**	1	3	3	1	-
**HBO + IL group (n = 8)**	2	2	3	1	-
**Control group (n = 8)**	^-^	1	2	2	3*
**Sham group (n = 8)**	3	3	2	-	-

*Significantly higher than the treatment groups by Chi-square test (*P* <0.05). HBO: hyperbaric oxygenation; IL: iloprost.

## Discussion

The aim of the present study was to examine the potential protective effects of HBO and IL on lung damage induced by limb I/R. Our main finding was that both HBO and IL attenuate lung injury. Furthermore, combined therapy of HBO and IL was shown to be more effective than either treatment given singly.

Although it is not clear why the lungs are more susceptible to injury than other remote organs (kidney, heart, liver and so on) after I/R of the lower limbs, we know that humoral mediators activate polymorphonuclear leukocytes, lead to aggregation at the lung capillaries, and adhere to the vascular endothelium, activating secretion of lysosomal enzymes that injures the lungs [15]. It has been shown that inflammatory cells like macrophages and mast cells in lungs produce inflammatory mediators and exaggerate reperfusion injury [16]. Lung injury after lower limb I/R causes pulmonary dysfunction characterized by interstitial edema and this leads to sustained ventilator and inotropic assistance and causes considerable mortality and morbidity. In ischemic injury, the basic therapy is surgical. Various drugs (aprotinin, alpha tocopherol, L-arginine, tacrolimus, ascorbic acid and others) were shown to be experimentally and clinically effective in reducing lung injury after lower limb ischemia reperfusion [17,18]. The reduction or prevention of neutrophil activation and sequestration would reduce the incidence of IRI lung injury. This hypothesis has been supported by studies in which it has been shown that the depletion of neutrophils ameliorates IRI lung injury [19].

The beneficial effects of HBO may be attributable to its ability to reduce neutrophil activation and sequestration in IRI. Studies in animal models have shown that HBO enhances fibroblast replication and collagen formation in scar tissue, improves neovascularization of ischemic tissue and decreases edema [20,21]. HBO has also been shown to reduce leukocyte adherence to venular endothelium in I/R of skeletal muscle, suggesting a reduction in the inflammation of the postischemic tissue [22]. The mechanisms behind this effect are not known, but the limited amount of available experimental data indicate that HBO, directly or indirectly, interacts with mediators that control the local inflammatory response [23]. Because HBO has been shown to exert anti-inflammatory effects in experimental skeletal muscle ischemia, it could have anti-inflammatory effects in other types of inflammation as well, although the most probable explanation for the reduction in the local inflammation in ischemia is a reduction in local ischemic tissue damage. In an intestinal IRI model, rats underwent 90 min of reperfusion after 2 h of ischemia. HBO was administered during the reperfusion period and a significant reduction in lung neutrophil sequestration was observed [8].

The beneficial effect of HBO on lung neutrophil sequestration may be achieved when it is administered during either the period of ischemia or the reperfusion. Rossman *et al.* reported that perfusing the lumen of the intestine with oxygenated perfluorocarbon during a period of ischemia reduced mucosal injury and associated lung injury [24]. Ueno *et al.* showed that early posthepatectomy HBO treatment decreased neutrophil activation and improved the outcome [25]. Convincingly, HBO should be administered as early as possible during I/R to obtain a favorable effect. In our study, both HBO and IL therapies were administered from the beginning of ischemia and continued during the reperfusion period.

There is disagreement concerning HBO therapy in IRI, due to the hypothesis that HBO can cause an increase in reactive oxygen derivatives, which can lead to tissue damage by lipid peroxidation when present in excess [26]. However, Chen *et al.* reported that HBO reduces lipid peroxidation in hepatic ischemia reperfusion [27]. Similarly in another study it was pointed out that HBO therapy at an appropriate pressure and within the appropriate time period reduces free oxygen radicals in animals [28]. It was noted that HBO therapy is safe using a standard protocol, with a pressure of not more than 3 atm and a therapy session under 120 minutes. The protective effect of HBO in this study shows that it suppresses the specific enzymes that catalyze lipid peroxidation.

Iloprost, a stable prostacyclin analog, acts as a membrane stabilizer and inhibits the function of neutrophils, which are potential mediators of I/R injury. Iloprost also decreases white blood cell aggregation and adhesion to the vascular endothelium, superoxide radical production from stimulated canine and human neutrophils, and free radical formation in myocardium subjected to ISI [29]. Iloprost had demonstrated its protective effects when infused during the clamping period in previous studies [30,31]. We obtained the dosage of the agent from a study performed by Katircioglu *et al.*[30]. In that study, administration was started at a lower rate before the aortic occlusion, and increased to the definitive dose of 25 ng/kg/min. Instead of doing this, we started directly at the definitive level to obtain the maximal physiologic effect before aortic occlusion. We did not perform a separate dose–response effect experiment. IL infusion was administrated during 60 min of ischemia and 60 min of reperfusion in the treatment groups.

In the present study, plasma MDA levels, serum LDH levels and arterial blood gas were measured to evaluate the I/R damage. Clinical research in the area of lipid peroxidation has been hampered by the lack of a valid biomarker. One of the most frequently used biomarkers providing an indication of the overall lipid peroxidation level is MDA [32]. MDA, a more stable and longer-living product of lipid peroxides, is often assayed as reflecting lipid peroxidation level. MDA is a marker that increases during reperfusion as an indicator of free radicals. Plasma and lung MDA levels in the control group were found to be significantly higher in the reperfusion period.

The involvement of neutrophils in I/R lung injury is important [8]. Neutrophils are activated during ischemia, and their adhesiveness and rigidity increase. During reperfusion, activated neutrophils are trapped within the lung and adhere to endothelial cells, generating oxidants and proteases [33]. Many mediators induce neutrophil activation, including TNF-α. The involvement of TNF-α in IRI is evidenced by two facts, that the production of TNF-α increases in IRI [34,35] and that the blockade of TNF-α activity reduces I/R-induced lung injury [35]. Yang *et al.* hypothesized that the suppressing effect of HBO on TNF-α production contributes to a reduced I/R-induced acute lung injury [36]. Our results support their hypothesis. Compared with the control group, serum TNF-α levels in the treatment groups and the sham group were found to be significantly lower in the reperfusion period. The histological lung injury score of the combined therapy group (HBO + IL) was not statistically different from the single therapy groups. However, the score in the HBO + IL group was (although statistically not significant) better than both the HBO group and IL group.

When the hemodynamic data were evaluated, no significant changes were seen in the vital signs of the animals. In the present study, the arterial blood gas samples were collected in the reperfusion period. Products of anaerobic metabolism enter the systemic circulation following the unclamping of the aorta. The level of CO_2_ released from ischemic tissue may increase during the clamping [37]. Because the blood circulation and oxygen delivery to the tissues decrease, the cellular metabolism supplies the required energy from the high-energy phosphates and lactic anaerobic method. This in turn causes increases in the lactate levels. Lactate levels can increase rapidly in the blood together with the release of the clamp [32]. The serum LDH level in the control group was found to be significantly higher in the reperfusion period.

## Conclusion

The present study showed that both HBO and IL reduce lipid peroxidation. Furthermore, combined therapy of HBO and IL was shown to be more effective than either treatment given alone. We also hypothesize that the combined effect may be related to the IL minimizing the side effects of HBO with its antioxidant property, and strengthening the effect of HBO by lowering lipid peroxidation.

## Abbreviations

ANOVA: Analysis of variance; CPK: Creatine kinase; CO2: Carbon dioxide; HBO: Hyperbaric oxygen; IL: Iloprost; I/R: Ischemia and reperfusion; IRI: Ischemia/reperfusion injury; K: Potassium; LDH: Lactate dehydrogenase; MDA: Malondialdehyde; Na: Sodium; pCO2: Partial pressure of carbon dioxide; pO2: Partial pressure of oxygen; SD: Standard deviation; TNF-α: Tumor necrosis factor alpha.

## Competing interests

The authors declare that they have no competing interests.

## Authors’ contributions

SB designed the study, wrote the manuscript, and killed the rabbits. GI designed the study. YY participated in the animal care and housing, and killed the rabbits. ZD participated in the animal care and housing, and killed the rabbits. LT carried out the pathological and histological study and evaluation. HK performed the statistical analysis. SOK participated in data collection and statistical analysis. SE participated in data collection. ES participated in study design. All authors read and approved the final manuscript.
